# What is the effect of the early follicular phase FSH/LH ratio on the number of mature oocytes and embryo development?

**DOI:** 10.3906/sag-1910-234

**Published:** 2020-04-09

**Authors:** Özgür ARAT, Derya DEVECİ, Zehra Sema ÖZKAN, Sevim TUNCER CAN

**Affiliations:** 1 Department of Obstetrics and Gynaecology, Fırat University School of Medicine, Elazığ Turkey; 2 Fırat University School of Health Services, Elazığ Turkey; 3 Department of Obstetrics and Gynaecology, Kırıkkale University School of Medicine, Kırıkkale Turkey

**Keywords:** FSH/LH ratio, mature oocyte, pregnancy rate, live birth rate

## Abstract

**Background/aim:**

Basal level of follicle stimulating hormone (FSH), luteinizing hormone (LH), estradiol (E2), and antral follicle count are used as predictors of ovarian reserve before starting ovulation induction. We aimed to investigate the predictor potential of early follicular phase FSH/LH ratio on controlled ovarian hyperstimulation-intracytoplasmic sperm injection (COH-ICSI) cycle outcomes.

**Materials and methods:**

This retrospective cohort study was conducted with 648 COH-ICSI cycles performed between 2012 and 2014. Cycles were classified according to their basal FSH/LH ratio, group 1(G1) = FSH/LH ratio < 2, N = 473 and group 2(G2) = FSH/LH ratio ≥ 2, N = 175. Demographic characteristics and stimulation parameters were evaluated. Retrieved total oocyte count (TOC), mature oocyte count (MOC), transferred embryo number, and pregnancy results were obtained and transferred to computer by SPSS 21.0 programme.

**Results:**

TOC and MOC of G1 were significantly higher than those of G2. The total gonadotrophin doses of G2 were significantly higher than G1. There was no significant difference between groups for transferred embryo number. Pregnancy and live birth rates were similar in both groups.

**Conclusion:**

In our population, increased FSH/LH ratio did not affect the rates of pregnancy and live birth negatively.

## 1. Introduction

The outcome of in vitro fertilization (IVF) is strongly dependent upon ovarian response to gonadotropin stimulation [1]. Ovarian stimulation can delivery 2 unpleasant results as either hyper or low response [2]. Due to these conditions, ovarian response prediction before stimulation is important for clinicians. Several methods can be used to predict ovarian stimulation results. Antral follicle count (AFC), day 3 (D3) follicle stimulating hormone (FSH) level, D3 estradiol (E2) level, anti-Müllerian hormone (AMH) level are the most used parameters [3]. High FSH levels or decreased AMH levels could predict the poor response for gonadotrophin stimulation but could not foretell for fecundity [4]. 

There is a negative correlation between female’s chronological age and ovarian reserve. The first finding of ovarian aging is a prominent increment in FSH levels compared to luteinizing hormone (LH) levels. This increment makes the FSH/LH ratio increased [5]. Increased FSH/LH ratio is a new method to determine the cycle outcome before starting stimulation [6]. Researchers reported that increased FSH/LH ratio can predict decreased ovarian reserve and lower pregnancy outcomes [7–9].

In this study we aimed to compare the cycle outcomes of patients’ experienced controlled ovarian hyperstimulation-embryo transfer (COH-ET) with either FSH/LH ratio higher than 2 or not.

## 2. Materials and methods

This retrospective cohort study was conducted with 648 COH-ET cycles performed between October 2012 and October 2014 after approval of local ethical committee (2014/13-04). Preparation of all patients before stimulation consisted of detailed anamnesis, physical examination, transvaginal ultrasonography, day 3 (D3) hormone profile [follicle stimulating hormone (FSH), luteinizing hormone (LH), oestradiol (E2), progesterone (P4), thyroid stimulating hormone (TSH), prolactin], semen analysis, hysterosalpingography or hysteroscopy. Antral follicle count (AFC) were determined on day 2/3 before starting stimulation. COH was performed with long GnRH agonist, GnRH antagonist, and micro dose flare-up protocols as appropriate.

The FSH/LH ratio cut-off is changing among studies, but the most used cut-off is 2 [1,3]. Due to this condition, we determined cut-off ratio as 2 and cycles were divided into 2 groups according to D3 FSH/LH ratio. Group 1 (G1) was consisted of 473 cycles with FSH/LH ratio < 2 and Group 2 (G2) was consisted of 175 cycles with FSH/LH ratio ≥ 2. 

GnRH agonists were initiated on luteal phase and gonadotropin stimulation was started on day 2/3 of the proceeding cycle. Gonadotropin doses [recombinant FSH (rFSH), human menopausal gonadotropin (HMG) or both] were determined according to age, FSH level, and AFC of women. In antagonist cycles, gonadotropin stimulation was started on day 2/3 of fresh cycle and when the leading follicle reached 12 mm in diameter or blood E2 level reached 300 pg/mL, cetrorelix or ganirelix were started. In micro dose flare-up protocol, oral contraceptive pill was started on day 2 of previous cycle for 21 days and on day 1 of proceeding cycle GnRH agonist was initiated (subcutaneous leuprolide acetate 40 µg/daily). On day 2 of cycle, exogenous gonadotropins were started. When at least 3 follicles reached 17 mm in diameter, recombinant human chorionic gonadotropin (hcg) was administered for final maturation. Both of GnRH antagonists and agonists were continued until the day of hcg injection. Oocyte pick-up (OPU) was performed at 34–36 h after hcg injection. 

In embryology, mature oocytes were inseminated by intracytoplasmic sperm injection (ICSI) after cumulus separation. Fertilization was defined as the observation of 2 pronuclei 24 h after ICSI. Embryo transfer (ET) was performed on day 3 with single cleavage embryo or on day 5 with single blastocyst. Luteal support was done with daily application of vaginal 8% progesterone gel and intramuscular 50 mg progesterone. Pregnancy was checked with blood beta hcg test 14 days after ET. Blood beta hcg test was repeated 2 days later for confirming the healthy increment. Transvaginal ultrasonography was performed for visualization of gestational sac 10 days later. Ongoing pregnancy was defined as the presence of more than 24 weeks of gestation with a live foetus. Implantation rate (ImR) was calculated as the ratio of gestational sac number/transferred embryo number. Fertilization rate (FR) was calculated as the ratio of fertilized oocyte number/mature oocyte number. Pregnancy rate (PR) was calculated as the ratio of beta hcg test positivity/transferred embryo number.

Age, body mass index (BMI), infertility aetiology, D3 FSH-LH-E2 levels, AFC, stimulation protocol type, amount of the used gonadotropins, E2 and P4 levels on the day of hcg injection, retrieved total oocyte count (TOC), mature oocyte count (MOC), fertilised oocyte count (FOC), transferred embryo number, and pregnancy results were recorded from patient files. Primary outcome of this study is the comparison of mature oocyte number and fertilisation rate between groups. Secondary outcome is the comparison of implantation rate and live birth rates between groups. 

Statistical analysis was performed with SPSS 21.0 version (IBM Corp., Armonk, NY, USA). Comparison of continuous variables between groups were done with student t-test or Mann–Whitney U test according to distribution normality of data. Comparison of categorical variables were done with chi-square test or Fisher exact test where applicable. For investigation of possible relation and interaction, correlation and regression analysis were performed respectively. P-value smaller than 0.05 was accepted as statistically significant.

## 3. Results

The demographic characteristics of groups are presented on Table 1. The mean age, infertility duration, BMI and D3 FSH level of G1 were significantly lower than those of G2. AFC of G1 was significantly higher than G2. The infertility aetiologies of groups are presented on Table 1. Decreased ovarian reserve and unexplained infertility were significantly lower in G1 compared to those in G2, but anovulation rate was significantly higher in G1 than G2.

**Table 1:** Demographic characteristics of groups.

	G1 (FSH/LH < 2)N = 473	G2 (FSH/LH ≥ 2)N = 175	P-value
Age (years)	30.7 ± 5.2	33.9 ± 5.5	<0.01
Infertility period (years)	5.9 ± 4.2	6.9 ± 4.8	0.02
BMI (kg/m2)	25.3 ± 4.3	26.3 ± 4.6	0.02
Previous cycle number	1.5 ± 0.9	1.7 ± 1.2	0.6
D3FSH (mIU/mL)	6.4 ± 4.4	9.1 ± 6.4	<0.01
AFC	10.1 ± 7.4	6.5 ± 5.8	<0.01
TSH (mIU/mL)	1.7 ± 1.1	1.5 ± 0.9	0.1
Infertility aetiology (%)			
-Male factor	38.1	32	0.1
-Tubal factor	10.1	5.5	0.06
-Anovulation	17.5	3.4	0.04
-Endometriosis	7.7	9.8	0.14
-Decreased ovarian reserve	9.7	22.9	0.03
- Unexplained infertility	17	26.4	0.04

Note: Values are presented as mean ± SD and n (%).

The stimulation characteristics of groups are presented on Table 2. While the GnRH agonist protocol rate was significantly higher in G1 than G2, the remaining protocol rates did not show significant difference between groups. Total and start HMG doses of G1 were significantly lower than G2. Oestradiol level on hcg day was significantly higher in G1 compared to that in G2. TOC, MOC, and FOC were significantly higher in G1 compared to those in G2. There was no significant difference between groups for parameters of FR, PR, ImR, and transferred embryo number.

**Table 2:** Stimulation and embryology characteristics of patients.

	G1 (FSH/LH < 2)N = 473	G2 (FSH/LH ≥ 2)N = 175	P-value
Pituitary suppression type (%)-antagonist -agonist -micro dose flare-up	63.3370.7	71.6280.4	0.060.010.12
Oestradiol level on hcg day (pg/mL)	2305.1 ± 1250.1	1656.4 ± 1144.8	<0.01
Endometrial thickness on hcg day (mm)	10.4 ± 2.3	10.1 ± 2.5	0.1
Progesterone level on hcg day (ng/mL)	0.9 ± 0.4	0.8 ± 0.4	0.23
Stimulation duration (day)	9.0 ± 1.8	9.3 ± 1.9	0.06
HMG start dose (IU)	76.9 ± 146.8	168.4 ± 199.4	<0.01
FSH start dose (IU)	251.9 ± 124.7	245.7 ± 173.3	0.3
HMG total dose (IU)	582.0 ± 1045.4	1236.1 ± 1377.5	<0.01
FSH total dose (IU)	1831.6 ± 1016.5	1749.6 ± 1342.3	0.6
Total oocyte number	13.1 ± 7.9	8.8 ± 6.4	<0.01
Mature oocyte number	9.7 ± 6.7	6.5 ± 4.6	<0.01
Fertilized oocyte number	7.1 ± 5.1	4.9 ± 3.7	<0.01
Mature/total oocyte rate (%)	73	76	0.12
Fertilization rate (%)	77	79	0.4
Implantation rate (%)	66	58	0.1
Transferred embryo number	1.9 ± 0.7	1.8 ± 0.6	0.3
Pregnancy rate (%)	37.6	35.4	0.4

Note: Values are presented as mean ± SD and percentage.

The pregnancy outcomes are presented on Table 3. There were no differences between groups for parameters of live birth rate and abortion rate.

**Table 3:** Pregnancy outcomes.

	G1 (FSH/LH < 2)(N = 178)	G2 : (FSH/LH ≥ 2)(N = 62)	P-value
Chemical abortion	36 (21%)	9 (16%)	0.72
Missed abortion	29 (16%)	12 (20%)	0.24
Ectopic pregnancy	2 (0.5%)	2 (0.5%)	0.16
Preterm delivery	3 (0.5%)	0	0.55
Live birth	108 (62%)	39 (63.5%)	0.85

Note: Values are presented as n (percentage).

Correlation (R = 0.08, P = 0.02) and regression analysis (OR = 0.6, 95% CI = 0.188–0.222, P < 0.01) revealed no relation between FSH/LH ratio and mature/total oocyte ratio. There was no relation between FSH/LH ratio and rates of fertilization and implantation.

## 4. Discussion

In this study we observed that increased FSH/LH ratio has no detrimental effect on pregnancy outcomes of infertile women. Increased FSH/LH ratio brings the costs of increased gonadotropin doses for stimulation with decreased total collected oocyte count. Fertilisation, implantation, and pregnancy rates of women with increased FSH/LH ratio did not show difference from women with normal FSH/LH ratio. Our results showed that decreased ovarian reserve did not have negative influence on pregnancy outcomes. In our study, we determined the live birth rate of our population and we observed that high FSH/LH ratio did not show negative impact on live birth rate.

Low ovarian response despite appropriate ovarian stimulation is an undesirable result of IVF programme [10]. The underlying causes of ovarian resistance to gonadotropin stimulation are still unclear. POSEIDON group (Patient-Oriented Strategies Encompassing IndividualizeD Oocyte Number) improved a new classification system in patients with decreased ovarian reserve or unexpected inappropriate ovarian response to exogenous gonadotropins [11]. The application of POSEIDON classification system with retrospective analysis would be more appropriate for prognosis evaluation [12]. AMH estimation, AFC/TOC ratio, FSH/LH ratio are the most recent methods that are used to predict stimulation outcome [13,14]. In our study, we compared the results of cycles between women with increased FSH/ LH ratio and normal. We observed decreased total and mature oocyte counts in women with increased FSH/LH ratio. But interestingly, this decrement did not show negative impact on pregnancy and live birth rates. Khan et al. compared infertile and fertile women for age related ovarian reserve decrement among the population under 40 years. They observed no significant difference for serum AMH levels and AFC between infertile and fertile women [15]. Similar to our results, Prasad et al. observed increased gonadotropin doses requirement with decreased MOC on women with FSH/LH ratio higher than 2. However contrary to our results, Prasad et al. reported lower pregnancy rates on women with increased FSH/LH ratio [7]. Zhen et al. investigated the outcomes of 472 IVF-ET cycles belong to 426 poor ovarian response patients. They detected significantly lower clinical pregnancy rate in women aged >40 years than in those aged < or = 40 years [16]. 

Ho et al. used the cut-off value of 3 for FSH/LH ratio and they observed that women with FSH/LH ratio bigger than 3 revealed less mature oocytes [17]. Johnson et al. reported that increased FSH/LH ratio despite normal basal FSH level pointed to decreased ovarian reserve and poor response [18]. Liang et al. compared basal FSH/LH ratio between cycles with cancellation or not. They observed significantly increased FSH/LH ratio in the cycles ended with cancellation [19]. Mukherjee et al. compared the cycle outcomes of 74 patients either with FSH/LH ratio bigger than 3.6 or not. They observed significantly lower total oocyte count and increased cycle cancellation rate in the group with FSH/LH ratio bigger than 3.6 [20]. Liu et al. investigated retrospectively 297 cycle outcomes of women either with FSH/LH ratio bigger than 2 or not. They reported increased cycle cancellation rate and lower pregnancy rate in women with FSH/LH ratio bigger than 2 [21]. Rehman et al. evaluated the cycle outcomes of 282 women retrospectively. They classified women into 2 groups according to median FSH/LH ratio of their population. They compared cycle outcomes between women with FSH/LH ratio bigger than 1.26 or not. They reported lesser oocyte and embryo quality in women with FSH/LH ratio bigger than 1.26 [3]. There was no consensus among the researchers about the FSH/LH ratio to predict the stimulation outcome. In our study, we excluded the cycles ended with cancellation. Due to this criterion, we did not make a comment for cycle cancellation rate on the basis of increased FSH/LH ratio.

Huang et al. researched the factors affecting live birth rate with a population consisted of 2277 IVF/ICSI-ET cycles. They observed significant differences between cycles ended either with live birth or not for the parameters of maternal and paternal age, body mass index, poor ovarian response, TOC, number of transferred embryos. They emphasized that maternal age and number of embryos transferred were independent factors affecting live birth rate [22]. Our results differed from those of Huang et al. This difference may arise from our small population. In a multicentre study, the researchers investigated the effect of TOC on cumulative live birth rate of patients experienced frozen-thawed ET after fresh cycle. They observed that the probability of live birth increased from 7 oocytes [23]. In another study, investigators analysed the impact of ICSI on cumulative live birth rate among couples without male factor infertility. The median time spent by couples from oocyte pick-up to live birth was similar between IVF and ICSI cycles [24]. Interestingly Rao et al. observed that physically active women experienced higher live birth rates compared to physically inactive women in IVF/ICSI cycles [25].

Orvieto et al. compared cycle outcomes between women stimulated with either HMG or rFSH on a population with FSH/LH ratio bigger than 2. In HMG group, they observed significantly higher number of top-quality embryos and higher implantation and clinical pregnancy rates compared to those in rFSH group [26]. In our study, the women with FSH/LH ratio bigger than 2 received more HMG compared to women with FSH/LH ratio smaller than 2. This treatment modification might improve pregnancy outcomes in our population. Barroso et al. investigated cycle outcomes of infertile women with either FSH/LH ratio bigger than 3 or not. After adjustment for transferred embryo number and age, women with FSH/LH ratio bigger than 3 showed significantly lower MOC, implantation, and pregnancy rates compared to women with FSH/LH ratio smaller than 3 [1]. In our study clinical pregnancy and live birth rates of women with FSH/LH ratio bigger than 2 did not show significant difference from those of women with FSH/LH ratio smaller than 2. 

In conclusion, in our population, increased FSH/LH ratio did not affect the rates of pregnancy and live birth negatively. Expanded and prospectively organised studies are needed to discuss our results.

## Acknowledgments

We thank staff of Fırat University IVF Unit for their support during our study.
